# The validity of single-item measures of health-related quality of life across groups differing in acute respiratory symptom severity

**DOI:** 10.1007/s11136-024-03694-0

**Published:** 2024-08-03

**Authors:** Adam B. Smith, John E. Ware, Patricia Aluko, Anuradha Kulasekaran

**Affiliations:** 1 Leeds Institute for Cardiovascular & Metabolic Medicine, University of Leeds, Leeds, UK; 2 John Ware Research Group Inc, Watertown, USA; 3 Reckitt, Global Medical Affairs – Respiratory, Slough, UK; 4 Reckitt, Data Analytics and Research Insights, Slough, UK

**Keywords:** QGEN-8, HRQOL, Acute respiratory, Chronic co-morbid conditions, SF-36, Cough, Sore throat

## Abstract

**Purpose:**

Practical considerations precluding health-related quality of life (HRQOL) monitoring in population and clinical research have spawned development of improved items for more brief surveys of frequently measured HRQOL outcomes. The aim of this study was to validate the use of the Quality of Life General (QGEN-8), a shorter 8-item alternative to the longer 36-item short form (SF)-36 Health Survey for measuring the same eight HRQOL domains across groups of adults with varying severity of acute respiratory symptoms, such as cough and sore throat.

**Methods:**

National Opinion Research Center (NORC) representative probability (*N* = 1,648) and supplemental opt-in (*N* = 5,915) U.S. adult samples were surveyed cross-sectionally online in 2020. Parallel analyses compared QGEN-8 and SF-36 estimates of group means for each of eight matching profile domains and summary physical and mental scores across groups differing in severity of acute symptoms and chronic respiratory conditions using analysis of covariance (ANCOVAs) controlling for socio-demographics and presence of chronic respiratory conditions.

**Results:**

In support of discriminant validity, ANCOVA estimates of QGEN-8 means with SF-36 estimates revealed the same patterns of declining HRQOL with the presence and increasing severity of symptoms and chronic condition severity.

**Conclusion:**

QGEN-8^®^ shows satisfactory validity and warrants further testing in cross-sectional and longitudinal population and clinical survey research as a more practical method for estimating group differences in SF-36 profile and summary component HRQOL scores.

**Supplementary Information:**

The online version contains supplementary material available at 10.1007/s11136-024-03694-0.

## Abstract (plain language summary)

Upper respiratory tract infections (URTI) with symptoms such as cough and sore throat are highly prevalent and negatively impact on health-related quality of life (HRQOL). Existing instruments that comprehensively measure HRQOL are lengthy, potentially increasing respondent burden and restricting their use in clinical studies and research. The aim of this study was to evaluate whether eight newly constructed survey items, the QGEN-8®, measure the same HRQOL outcomes as the 36-item SF-36 Health Survey well enough to serve as a more practical alternative for purposes of detecting the physical and mental HRQOL effects on differing severity of acute URTI symptoms, specifically cough and sore throat. The results showed that the QGEN-8® was psychometrically sound and able to differentiate between different levels of URTI symptoms, even in cases where respondents had chronic respiratory conditions. This indicates that the briefer QGEN-8® with 75% shorter response time is able to provide HRQOL measurements comparable to those derived from lengthier instruments thereby lending itself more readily to use in clinical studies and research of URTI symptoms, such as cough and sore throat.

**Supplementary Information:**

The online version contains supplementary material available at 10.1007/s11136-024-03694-0.

## Introduction

Acute upper respiratory tract infections (URTIs) are highly prevalent accounting for up to 43% of all global diseases and infections with more than 17 billion cases [1]. URTIs are characterised by swelling and irritation of the upper airways. Most individuals present with mild symptoms such as cough, sore throat, runny nose, headache, and nasal congestion lasting from 7 days to 3 weeks. Although often self-limiting, symptoms are commonly perceived as unpleasant, affecting physical, psychological, emotional, social and general well-being [2, 3].

URTIs while non-fatal may, nevertheless, impact health-related quality of life (HRQOL) and work productivity. Global estimated costs associated with the economic burden of URTIs range from US$1 billion to US$22.5 billion in terms of total productivity loss [4–7] with underlying factors reflecting 10% work absences and 20% of workers being unfit for work due to URTIs [8, 9].

The acute symptoms of URTI, such as cough and sore throat, have been shown to impact negatively on HRQOL [10–13]. However, the patient-reported outcome measures (PROs) – both URTI- symptom-specific [10, 13] as well as generic instruments used to capture HRQOL – tend to be lengthy [11, 12]. Given the prevalence of URTI [1] there is, therefore, a need to develop shorter, more efficient PROs that are more feasible for both clinical studies and routine clinical practice to capture URTI symptom burden as well as potential treatment benefits.

Quality of Life General (QGEN)-8® single-item-per-domain measures [14, 15] were constructed anew to better represent short-form (SF)-36 domain content [16] and increase score range without using multiple items per domain. In comparison with SF-36®, QGEN-8 items® [15] have been shown to reduce respondent burden (by 75%) and significantly reduce score distribution ceiling effects while also maintaining construct validity for purposes of estimating group-level 8-domain profile, and physical (PCS) and mental (MCS) summary scores [15]. The current study is the first to compare the validity of QGEN-8® and SF-36® for purposes of capturing the HRQOL effects of group differences in the severity of acute respiratory symptoms.

The aim of this study, therefore, was to empirically evaluate the validity of QGEN-8® items in a head-to-head comparison with the SF-36-v2® multi-item scales and summary scores in terms of their validity in discriminating across groups differing in acute respiratory symptoms (cough and sore throat) among adults with and without a chronic respiratory condition.

## Methods

### Survey

The participant sample was drawn from the National Opinion Research Center (NORC) AmeriSpeak® [15] panel to represent the adult US population and supplemented with additional samples. This blended approach resulted in a true probability sample (*N* = 1,648) representing 97% of US household respondents and a supplemental opt-in sample (*N* = 5,915). These samples were combined to increase sample sizes particularly for the most severe (least frequent) symptom categories [17]. The survey was administered to participants on the internet (98.6%) or by telephone (1.3%) over a 3-month period from April to July 2020. All participants provided informed consent prior to survey. The survey was conducted in accordance with the guidelines of the American Association for Public Opinion Research (AAPOR) and was approved by the NORC Institutional Review Board (protocol number 20.05.29).

### Instruments

Participants completed two HRQOL instruments: the SF-36® and QGEN-8® [15, 16]. These two instruments have been shown using classical and modern psychometric methods to measure the same eight generic HRQOL domains: Physical Functioning, Role-Physical, Bodily Pain, General Health, Vitality, Social Functioning, Role-Emotional, and Mental Health, which are also used to estimate PCS and MCS scores (Supplementary Table 1). Thus, QGEN-8® is a brief single-item alternative for estimating the SF-36®, i.e., each of the domains on the longer version is represented by one new QGEN-8® item. All are scored positively and scores are transformed to have a mean of 50 and standard deviation (SD) of 10 in the 2020 US general population [18]. The responsiveness of the SF-36 to change in respiratory symptoms has been previously reported [12, 19, 20] further supporting the instruments use in the validation of the QGEN-8®.

### Statistical analysis

The validity of the QGEN-8® for use in URTI was evaluated through agreement with the SF-36, ability to detect change in symptom severity (including in the presence of respiratory comorbidities) as well as ability to identify clinically meaningful change. The processes applied to assess these criteria are described in detail below.

### Agreement

The level of agreement between the PCS and MCS from the SF-36® and QGEN-8® was investigated using Pearson’s product-moment correlations and Bland-Altman plots [21]. For the latter, mean scores on the PCS and MCS as derived from the SF-36® and QGEN-8®, respectively, were plotted against the mean differences of these scores. The percentage of data falling outside two standard deviations (*±* 2SD) of the difference was calculated to provide an assessment of the level of agreement. A cut-off of *≤* 5% of data falling within 2SD was assumed to be a good level of agreement.

### Acute respiratory symptoms

Continuous data were summarised using means and standard deviations. Participants recorded levels of symptoms (cough and sore throat) on a 4-point scale (“Not at all”, “several days”, “most of the days” and “nearly every day”). The ability of the QGEN-8® to discriminate between different levels of severity of acute URTI symptomatology was explored using one-way analysis of variance (ANOVA). *Post hoc* Bonferroni tests were applied to evaluate statistically significant differences between levels of symptoms for cough and sore throat.

Analysis of covariance (ANCOVA) was used to control for the main effects of comorbid conditions, which could cause acute symptoms and/or lower HRQOL. The ANCOVA models included a dummy variable for those chronic respiratory conditions (asthma and chronic obstructive pulmonary disease (COPD)), which were known to be sufficiently prevalent and known to impact on HRQOL and acute respiratory symptoms [22].

Performance of the four HRQOL summary measures (PCS, MCS, and the QGEN-PCS (ePCS) and QGEN-MCS (eMCS)) as well as for the SF-36® multi-item domain and the individual QGEN-8® items was compared using relative validity (RV) by calculating the F-ratios from the one-way ANOVA. The SF-36® F-ratios were used as the reference values: an RV < 1 would suggest the PCS was less valid relative to its QGEN counterpart. A 0.5SD was used to evaluate clinically meaningful group mean differences between symptom severity groups [23].

Data were analysed using R v4.03 [24]. A p value *≤* 0.05 was considered to be statistically significant.

## Results

### Participants

A detailed description of the study sample and SF-36® scores has previously been reported [12]. Briefly, a total of 6,660 participants (74% females) completed both the SF-36v2® and QGEN-8® questionnaire. A total of 6,654 (99.9%) and 6623 (99.4%) had severity data recorded for “Coughing” and “Sore Throat” respectively. Table 1 provides an overview of the sociodemographic details of all respondents (*N* = 6,660). The average age of this sample was 51.8 years (SD: 15.8, range: 18 to 100 years). Half the sample identified as White (50%); 26% as Black and 17% as Hispanic. A small majority had an undergraduate degree or above (54%); 27% had completed some college education. Approximately half the sample (51%) were in paid work either as an employee or self-employed with 8% retired and 10% unemployed.

**Table 1:** Socio-demographic characteristics

Characteristic	*N* = 6,660
*Gender (n, %)*	
Female	4,956 (74%)
Male	1,704 (26%)
**Age, years (mean, SD)**	51.8 (15.8)
*Ethnicity (n, %)*	
White	3,341 (50%)
Black	1,711 (26%)
Hispanic	1,123 (17%)
Asian	260 (4%)
Mixed	162 (2%)
Other	63 (1%)
*Education (n, %)*	
High school diploma or equivalent	940 (14%)
No high school diploma	276 (4%)
Some college	1,829 (27%)
Undergraduate degree or above	3,615 (54%)
*Employment status (n, %)*	
Employed / Self-employed	3,394 (51%)
Retired	1,851 (28%)
Other	767 (12%)
Unemployed	648 (10%)

SD, standard deviation

### Agreement between instruments

High Pearson product-moment correlations were observed between the QGEN-8® and SF-36® estimates for the two summary HRQOL measures: *r* = 0.82 (*p* < 0.001) for the Physical measures (PCS and ePCS) and *r* = 0.83 for the Mental Health (MCS and eMCS) measures (*p* < 0.001).

The mean Physical measure scores were 50.23 (SD: 9.97) and 50.04 (SD: 10.37) for the PCS and ePCS, respectively, with a mean difference of 0.19 (SD: 6.09). For the Mental Health measures these were 49.59 (SD: 10.32) and 51.41 (10.76), for MCS and eMCS, respectively; the mean difference was 1.81 (SD 6.22).

**Fig. 1:**
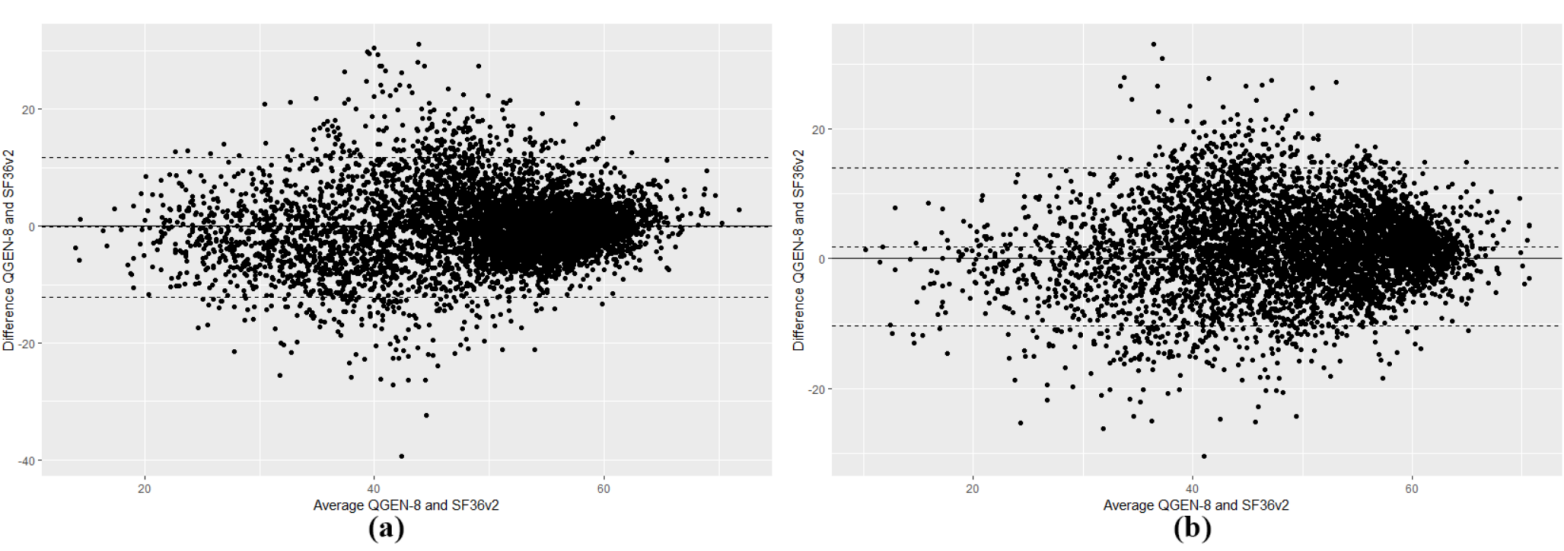
(**a**) Bland-altman plot for the Physical Component Summary (PCS). (**b**). Bland-Altman plot for the Mental Health Component Summary (MCS)

The Bland-Altman plots showed that only 6.2% and 3.3% of estimate differences fell outside the ± 2 SD threshold, respectively, for the summary Physical and Mental Health measures (Fig. 1a and b).

### Acute respiratory symptoms: ePCS and eMCS

**Fig. 2:**
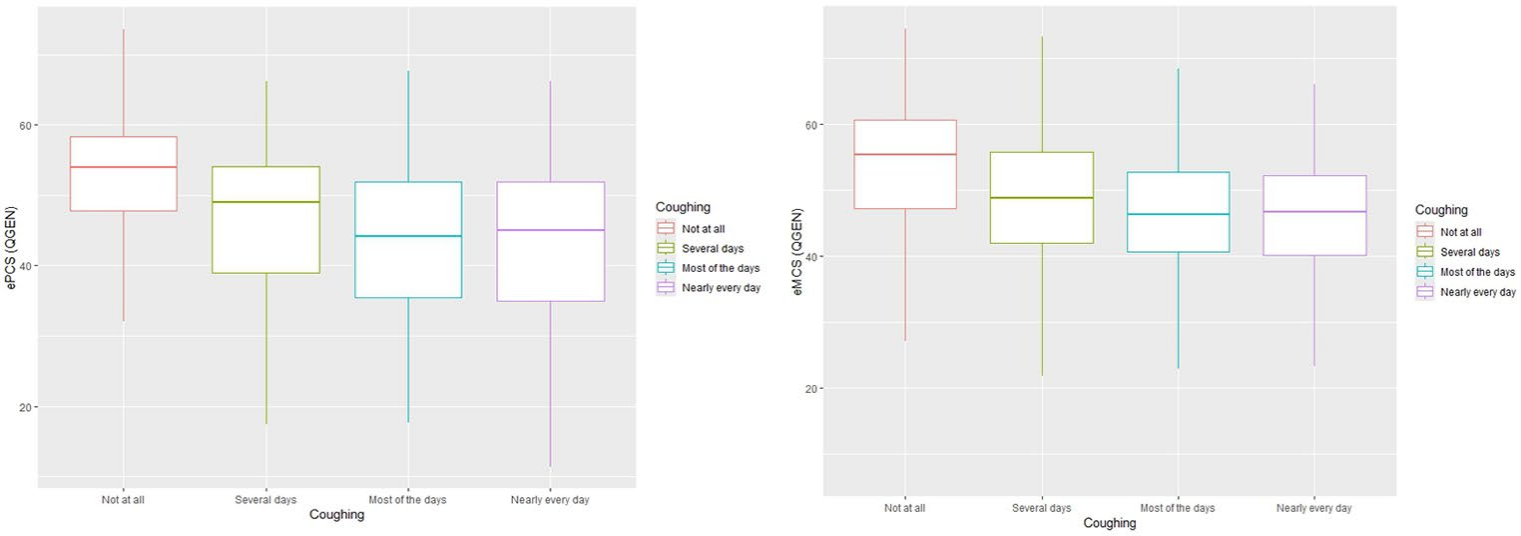
QGEN ePCS, eMCS and cough severity

**Fig. 3:**
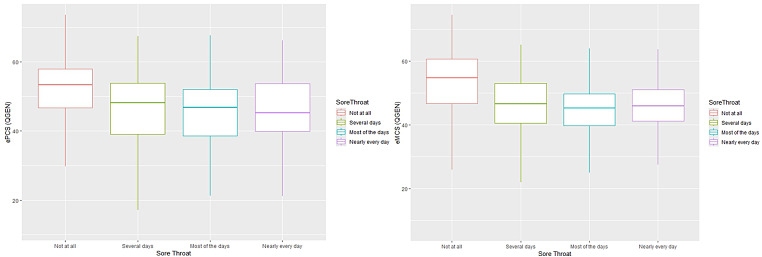
QGEN ePCS, eMCS and sore throat

Tables 2 and  3 shows the mean ePCS and eMCS scores for groups differing in cough and sore throat severity. As reported previously, the SF-36 detected substantial impact of symptom presence and progressive decline with increased symptom severity for both summary measures (Figs. 2 and 3). The largest differences were seen between the first and last response categories (“Not at all” and “Nearly every day”). For both symptoms these differences exceeded 0.5SD for both the ePCS and eMCS suggesting clinically meaningful differences. All these differences were statistically significant (Tables 2 and 3). For the QGEN-8®, the post hoc comparisons showed statistically significant differences for cough severity for all contrasts with the exception of the adjoining categories for the most severe symptoms for the ePCS (“Nearly every day” and “Most of the days”); this applied to both PCS and MCS for the SF-36 (Supplementary Tables 2a and b). For the QGEN-8® eMCS there was additionally no statistically significant difference between “Nearly every day” and “Several days” (*p* = 0.16). The pattern was slightly different for Sore Throat. Although the QGEN-8 ePCS/eMCS and SF-36 PCS/MCS were consistent within each instrument, three post hoc contrasts (“Not at all” versus the three other categories) were statistically significant for the ePCS / eMCS; whereas the PCS / MCS followed the same pattern as for Cough severity.

**Table 2:** Mean QGEN-8 and SF-36 component scores - cough

Cough severity	Not at all, *N* = 5,137^*1*^	Several days, *N* = 821^*1*^	Most of the days, *N* = 328^*1*^	Nearly every day, *N* = 253^*1*^	F-ratio	RV	*p*-value^2^
ePCS (QGEN)	51.5 (9.8)	46.2 (10.7)	43.0 (10.1)	43.5 (11.4)	164.7	1.6	< 0.001
PCS (SF-36)	51.9 (9.3)	46.1 (9.6)	41.8 (8.4)	41.7 (11.3)	263.5		< 0.001
eMCS (QGEN)	52.6 (10.5)	47.8 (10.9)	45.7 (10.2)	46.1 (10.2)	109.0	1.8	< 0.001
MCS (SF-36)	51.1 (9.9)	45.7 (10.1)	42.0 (8.9)	42.3 (10.9)	196.2		< 0.001

e/PCS, Physical Component Summary; e/MCS, Mental Health Component Summary; RV, relative validity

^*1*^ Mean (SD)

^*2*^ One-way analysis of variance (ANOVA)

Although significant F-ratios confirmed significant differences among groups for both symptoms for both methods, the RV values (Table 2 and 3) indicated that the validity of both the SF-36® PCS and MCS was greater relative to the QGEN-8© counterparts for both sore throat and cough.

**Table 3:** Mean QGEN-8 and SF-36 component scores – sore throat

Sore Throat severity	Not at all, *N* = 5,701^*1*^	Several days, *N* = 498^*1*^	Most of the days, *N* = 214^*1*^	Nearly every day, *N* = 126^*1*^	F-ratio	RV	*p*-value^2^
ePCS (QGEN)	50.7 (10.2)	46.0 (10.5)	45.0 (9.8)	45.9 (9.5)	59.03	2.6	< 0.001
PCS (SF-36)	51.2 (9.7)	45.5 (9.7)	41.5 (7.4)	41.8 (10.8)	153.5		< 0.001
eMCS (QGEN)	52.3 (10.7)	45.7 (10.3)	44.6 (8.2)	45.5 (8.1)	106.3	2.2	< 0.001
MCS (SF-36)	50.8 (10.0)	42.9 (9.6)	39.4 (6.4)	39.5 (7.8)	233.9		< 0.001

e/PCS, Physical Component Summary; e/MCS, Mental Health Component Summary; RV, relative validity

^*1*^ Mean (SD)

^*2*^ One-way analysis of variance (ANOVA)

**Fig. 4:**
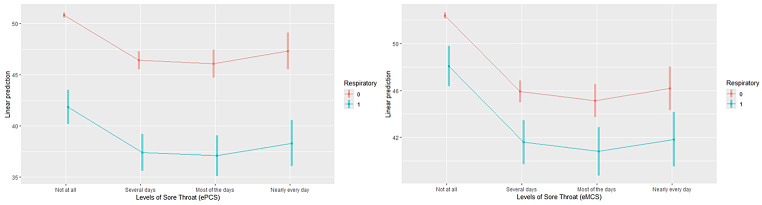
Estimated marginal means for ePCS and eMCS by sore throat and respiratory disease (Asthma and COPD)

**Fig. 5:**
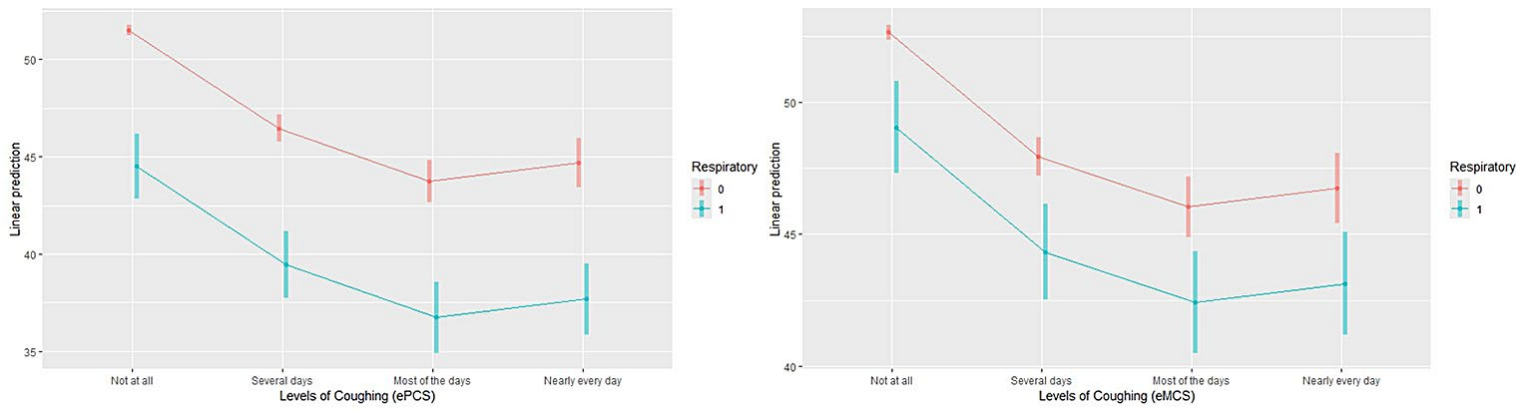
Estimated marginal means for ePCS and eMCS by coughing and respiratory disease (Asthma and COPD)

The same pattern was also observed when controlling for comorbid respiratory conditions (COPD and asthma) (Figs. 4 and 5). For both sore throat and cough, participants with comorbid respiratory conditions demonstrated significantly lower HRQOL scores on both the ePCS and eMCS, which decreased as symptom burden increased. These results (ANCOVA) were statistically significant for both Cough severity (ePCS, F(4,6534) = 142.3, *p* < 0.001; eMCS, F(4,6534) = 86.1, *p* < 0.001), and Sore Throat (ePCS, F(4,6534) = 73.5, *p* < 0.001; eMCS, F(4,6534) = 86.1, *p* < 0.001).

## Discussion

This study aimed to validate the QGEN-8® across groups of participants differing in levels of severity of URTI, including cough and sore throat symptoms, in comparison with results for the longer form SF-36®. These symptoms, particularly cough and sore throat, are critical to focus on as they are common to various respiratory conditions, including COVID-19 [25]. The criteria applied to evaluate validity included the correspondence of the QGEN-8® with the SF-36, ability to detect change, as well as the ability to clinically meaningful change. The results demonstrated a high level of correlation between the QGEN-8® ePCS and eMCS summaries and the corresponding SF-36 summaries, PCS and MCS. There was also a high degree of agreement between the QGEN-8® and the corresponding SF-36® components with the Bland-Altman analyses suggesting equivalence in measurement, particularly for the Mental Health Component score (eMCS). Both the physical and mental health components (ePCS and eMCS) of the QGEN-8® demonstrated a concomitant change as symptom severity increased for both cough and sore throat symptoms, particularly for three of the four levels of symptom severity, although less differentiation was observed between the two most severe categories (“Most of the days” and “”Nearly every day). Furthermore, clinically meaningful differences were observed between the most (“Nearly every day”) and least symptom burden (“Not at all”) for both cough and sore throat. These results were maintained even when controlling for chronic respiratory conditions such as asthma and COPD. The study therefore confirms the same pattern of results was detected using the QGEN-8© compared with the SF-36® [12].

These results underscore the potential of brief, easy to administer PROs, such as the QGEN-8®, to measure HRQOL for cough and sore throat symptoms in URTI. The current study findings demonstrated that with the standardized SF-36® norm-based scoring of response categories, the QGEN-8® single-items provided sufficiently accurate estimates of group means for the SF-36® PCS and MCS for surveying HRQOL across groups differing in the severity of acute respiratory symptoms. The QGEN-8® has been shown to reduce respondent burden significantly [15] whilst maintaining validity in relation to SF-36 scores and measurement accuracy in estimating differences across symptom severity groups, and as documented elsewhere [15], the QGEN-8 brevity was achieved while also significantly reducing US population single-item ceiling effects in comparison with other (SF-36, SF-12 and SF-8) measures of the same QOL domains. Therefore, it provides a more practical alternative for studying group-level differences in symptom and disease burden and treatment benefit in clinical studies and clinical practice.

Study limitations that are noteworthy include those inherent in cross-sectional survey research including: lack of tests of responsiveness to change, reliance on self-report for all study variables and order effects of different modules. Although such effects may have shifted results for some variables it is doubtful that these effects biased conclusions regarding the relative performance of the methods compared across identical groups differing in symptom severity. There were no statistically significant differences (or trend) observed between the two most severe symptom groups observed for both physical and mental outcomes using both methods (QGEN-8 and SF-36), which calls this particular criterion category distinction into question for both symptoms. Other respiratory-specific instruments make greater distinctions between response categories, for instance, through weighting [26], and the question for further evaluation is, therefore, whether there is a difference between “most” and “nearly every” day. Nevertheless, it remains that both methods studied agree with respect to this lack of difference. The current observed pattern of reduced power in detecting group differences using shorter measures is consistent with previous studies [15] and can be offset by larger samples.

It is hoped that the more practical QGEN-8® survey will facilitate longitudinal tests with larger samples to evaluate responsiveness to change over time to provide a better understanding of impact of HRQOL in URTI, particularly cough and sore throat. Given the greater degree of relative validity for the SF-36® PCS and MCS this instrument would lend itself to selection when greater accuracy is needed for interpretation of individual differences, for instance in clinical use; however, the results underline that the briefer QGEN-8® has validity for broader use in clinical trials and research.

## Conclusions

In conclusion, QGEN-8® single-item estimates of HRQOL domains show satisfactory validity for purposes of more practically estimating SF-36 summary PCS and MCS scores and using them to discriminate among groups of adults known to differ cross-sectionally in upper respiratory symptom severity. This shorter form warrants further testing in clinical trials or real world studies to evaluate reduced HRQOL burden from interventions for URTI symptom relief. QGEN-8® as a reduced respondent burden tool, should be assessed for impact on HRQOL in other short-term and long term conditions, longitudinally in population and clinical surveys.

## Electronic supplementary material

Below is the link to the electronic supplementary material.

Supplementary Material 1
