# Supplementary material for: The validity of single-item measures of health-related quality of life across groups differing in acute respiratory symptom severity
Source: Qual Life Res. 2024 Aug 3;33(10):2773–80. doi: 10.1007/s11136-024-03694-0 (PMC11452483; doi:10.1007/s11136-024-03694-0)
Supplement: Supplementary file 1 — Supplementary Material 1 [file 11136_2024_3694_MOESM1_ESM.docx]

**Supplementary Table 1. Summary comparison of QGEN-8 and SF-36 measures**

| **Dimensions/Domains** | **Number of**  **Items** | **Raw Score**  **Range^a^** | **Norm-based**  **Score Range^b^** | **Ceiling effects (%) ^c^** |
| --- | --- | --- | --- | --- |
|  |  |  |  |  |
| **Physical Function (PF)** |  |  |  |  |
| **QGEN-8** | 1 | 1-5 | 23.9-58.6 | 34.9 |
| **SF-36** | 10 | 10-30 | 16.4-72.4 | 37.1 |
| **Role-physical (RP)** |  |  |  |  |
| **QGEN-8** | 1 | 1-5 | 25.9-57.3 | 27.9 |
| **SF-36** | 4 | 3-15 | 16.4-68.2 | 48.8 |
| **Pain (BP)** |  |  |  |  |
| **QGEN-8** | 1 | 1-5 | 27.9-61.4 | 31.0 |
| **SF-36** | 2 | 10-20 | 19.8-58.5 | 45.3 |
| **General Health (GH)** |  |  |  |  |
| **GEN-8** | 1 | 1-5 | 25.4-65.4 | 6.9 |
| **SF-36** | 5 | 5-25 | 22.7-58.9 |  |
| **Social Functioning (SF)** |  |  |  |  |
| **QGEN-8** | 1 | 1-5 | 26.8-60.2 | 31.3 |
| **SF-36** | 2 | 2-10 | 21.4-62.3 | 50.1 |
| **Vitality (VT)** |  |  |  |  |
| **QGEN-8** | 1 | 1-5 | 26.3-59.1 | 2.5 |
| **SF-36** | 4 | 4-20 | 19.1-67.4 | 6.9 |
| **Role-emotional (RE)** |  |  |  |  |
| **QGEN-8** | 1 | 1-5 | 26.8-60.2 | 19.7 |
| **SF-36** | 3 | 3-15 | 23.5-69.1 | 47.5 |
| **Mental Health (MH)** |  |  |  |  |
| **QGEN-8** | 1 | 1-5 | 22.5-62.1 | 6.7 |
| **SF-36** | 5 | 5-25 | 13.2-56.8 | 8.8 |
| **Physical Summary (PCS)** |  |  |  |  |
| **QGEN-8** | 8 | N/A | 16.4-72.4 |  |
| **SF-36^b^** | 35 | N/A | 15.9-69.0 |  |
| **Mental Summary (MCS)** |  |  |  |  |
| **QGEN-8** | 8 | N/A | 16.4-68.2 |  |
| **SF-36** | 35 | N/A | 9.0-71.1 |  |

^a^ Theoretical and observed raw score ranges for each method were the same.

^b^ Norm-based score ranges are those observed in the representative probability sample.

Note: QGEN-8 norm-based scores for each domain were estimated by assigning the

2020 SF-36 population group mean score observed for each item response category for the

corresponding domain [15]. For estimations of summary physical and mental component

scores, the SF-36 original SF-36 factor score coefficients were applied for both methods [15].

c Ceiling effect percentages for the SF-36 are those for the best (lowest ceiling %) and were much higher for all other SF-36 items

**Supplementary Table 2a. Post hoc contrasts for the QGEN-8® ePCS and eMCS by Cough severity**

| **Coughing** | **ePCS** | | | **eMCS** | | |
| --- | --- | --- | --- | --- | --- | --- |
| **QGEN-8 / Category Contrasts** | **Difference** | **95%CI** | **p** | **Difference** | **95%CI** | **p** |
| Several days-Not at all | -5.25 | -6.25, -4.26 | p<0.001 | -4.82 | -5.86, -3.78 | p<0.001 |
| Most of the days-Not at all | -8.44 | -9.94, -6.94 | p<0.001 | -6.96 | -8.54, -5.38 | p<0.001 |
| Nearly every day-Not at all | -7.95 | -9.65, -6.25 | p<0.001 | -6.49 | -8.28, -4.71 | p<0.001 |
| Most of the days-Several days | -3.19 | -4.91, -1.46 | p<0.001 | -2.14 | -3.95, -0.33 | p<0.01 |
| Nearly every day-Several days | -2.70 | -4.59, -0.8 | p<0.01 | -1.67 | -3.67, 0.32 | p=0.16 |
| Nearly every day-Most of the days | 0.49 | -1.72, 2.7 | p=1 | 0.47 | -1.85, 2.79 | p=1 |
| **SF-36** | **PCS** | | | **MCS** | | |
| Several days-Not at all | -5.78 | -6.71, -4.85 | p<0.001 | -5.41 | -6.39, -4.43 | p<0.001 |
| Most of the days-Not at all | -10.10 | -11.52, -8.69 | p<0.001 | -9.07 | -10.56, -7.59 | p<0.001 |
| Nearly every day-Not at all | -10.22 | -11.82, -8.62 | p<0.001 | -8.82 | -10.5, -7.14 | p<0.001 |
| Most of the days-Several days | -4.32 | -5.95, -2.7 | p<0.001 | -3.67 | -5.37, -1.96 | p<0.001 |
| Nearly every day-Several days | -4.44 | -6.23, -2.65 | p<0.001 | -3.41 | -5.29, -1.53 | p<0.001 |
| Nearly every day-Most of the days | -0.12 | -2.2, 1.96 | 1 | 0.26 | -1.93, 2.44 | 1 |

**Supplementary Table 2b. Post hoc contrasts for the QGEN-8® ePCS and eMCS by Sore Throat severity**

| **Sore Throat** | **ePCS** | | | **eMCS** | | |
| --- | --- | --- | --- | --- | --- | --- |
| **QGEN-8 / Category Contrasts** | **Difference** | **95%CI** | **p** | **Difference** | **95%CI** | **p** |
| Several days-Not at all | -4.76 | -6.02, -3.5 | p<0.001 | -6.62 | -7.92, -5.32 | p<0.001 |
| Most of the days-Not at all | -5.72 | -7.6, -3.84 | p<0.001 | -7.71 | -9.64, -5.78 | p<0.001 |
| Nearly every day-Not at all | -4.83 | -7.27, -2.4 | p<0.001 | -6.84 | -9.34, -4.34 | p<0.001 |
| Most of the days-Several days | -0.96 | -3.17, 1.25 | p=1 | -1.09 | -3.36, 1.18 | p=1 |
| Nearly every day-Several days | -0.07 | -2.76, 2.62 | p=1 | -0.22 | -2.99, 2.55 | p=1 |
| Nearly every day-Most of the days | 0.89 | -2.14, 3.92 | p=1 | 0.87 | -2.25, 3.99 | p=1 |
| **SF-36** | **PCS** | | | **MCS** | | |
| Several days-Not at all | -5.66 | -6.85, -4.47 | p<0.001 | -7.98 | -9.19, -6.77 | p<0.001 |
| Most of the days-Not at all | -9.75 | -11.52, -7.98 | p<0.001 | -11.49 | -13.29, -9.69 | p<0.001 |
| Nearly every day-Not at all | -9.45 | -11.74, -7.16 | p<0.001 | -11.32 | -13.65, -8.99 | p<0.001 |
| Most of the days-Several days | -4.09 | -6.17, -2.02 | p<0.001 | -3.51 | -5.63, -1.4 | p<0.001 |
| Nearly every day-Several days | -3.79 | -6.32, -1.25 | p<0.001 | -3.34 | -5.92, -0.76 | p<0.005 |
| Nearly every day-Most of the days | 0.31 | -2.55, 3.16 | p=1 | 0.17 | -2.74, 3.08 | p=1 |
